# Supplementary material for: Elevation and land use shape soil entomopathogenic fungal communities in the Uluguru mountains, Tanzania: Insights from metagenomic and culture-based approaches
Source: PLoS One. 2026 May 11;21(5):e0348781. doi: 10.1371/journal.pone.0348781 (PMC13160300; doi:10.1371/journal.pone.0348781)
Supplement: S1 Table — (DOCX) [file pone.0348781.s001.docx]

**S1Table.** Two-way ANOVA for Diversity indices by Land use, Altitude and their interaction

| **Factor** | **P-values** | | | |
| --- | --- | --- | --- | --- |
|  | **Shanon** | **Simpson (1-D)** | **Richness** | **Fisher alpha** |
| Altitude | 0.9801 | 0.9739 | 0.7064 | 0.9752 |
| Land use | 0.1540 | 0.1477 | 0.4773 | 0.1515 |
| Altitude:Land use | 0.2241 | 0.1578 | 0.3029 | 0.3577 |
| Total | 0.7357 | 0.5274 | 0.0184 | 0.1059 |

Notes: P-values <0.05 are statistically significance different
