# Supplementary material for: Elevation and land use shape soil entomopathogenic fungal communities in the Uluguru mountains, Tanzania: Insights from metagenomic and culture-based approaches
Source: PLoS One. 2026 May 11;21(5):e0348781. doi: 10.1371/journal.pone.0348781 (PMC13160300; doi:10.1371/journal.pone.0348781)
Supplement: S2 Table — (DOCX) [file pone.0348781.s002.docx]

**S2Table.** PERMANOVA analysis across the elevations

| **group1** | **group2** | **R2** | **F-value** | **df1** | **df2** | **p-value** |
| --- | --- | --- | --- | --- | --- | --- |
| Low | Medium | 0.341 | 3.11 | 1 | 6 | 0.0858 |
| Low | High | 0.314 | 2.748 | 1 | 6 | 0.12915 |
| Medium | High | 0.208 | 1.577 | 1 | 6 | 0.1419 |
| Overall/Total |  | 0.399 | 1.776 |  |  | 0.06 |
