# Supplementary material for: Elevation and land use shape soil entomopathogenic fungal communities in the Uluguru mountains, Tanzania: Insights from metagenomic and culture-based approaches
Source: PLoS One. 2026 May 11;21(5):e0348781. doi: 10.1371/journal.pone.0348781 (PMC13160300; doi:10.1371/journal.pone.0348781)
Supplement: S3 Table — (DOCX) [file pone.0348781.s003.docx]

**S3Table.** Metadata showing sampling sites and GPS Coordinates for soil samples from different land use and altitudes

| **Sampling site** | **Altitude a.ms.l (m)** | **GPS coordinates** | **Land use type** |
| --- | --- | --- | --- |
| SUA | 527.9 | -6.849530, 37.656842 | Cultivated |
|  | 517.2 | -6.848870, 37.654420 | Cultivated |
|  | 518.5 | -6.850367, 37.654367 | Cultivated |
|  | 516.6 | -6.848767, 37.655817 | Cultivated |
|  | 525.0 | -6.849983, 37.656288 | Fallow |
|  | 517.6 | -6.851033, 37.653700 | Fallow |
|  | 516.3 | -6.850133,37.653017 | Fallow |
|  | 503.5 | -6.848867, 37.654417 | Fallow |
| LANGALI | 1090.2 | -7.055314,37.577066 | Cultivated |
|  | 1076.2 | -7.054615,37.576427 | Cultivated |
|  | 1082.0 | -7.054157,37.576531 | Cultivated |
|  | 1117.8 | -7.053379,37.574636 | Cultivated |
|  | 1083.7 | -7.055269,37.577056 | Fallow |
|  | 1081.2 | -7.054879,37.576117 | Fallow |
|  | 1122.2 | -7.054669,37.574478 | Fallow |
|  | 1117.7 | -7.054922,37.574417 | Fallow |
| NYANDIRA | 1708.6 | -7.083641,37.581752 | Cultivated |
|  | 1713.8 | -7.084386,37.583739 | Cultivated |
|  | 1729.0 | -7.085506,37.582032 | Cultivated |
|  | 1677.1 | -7.085360,37.580418 | Cultivated |
|  | 1667.2 | -7.084210,37.580531 | Fallow |
|  | 1688.8 | -7.083923,37.580606 | Fallow |
|  | 1728.2 | -7.085817,37.581457 | Fallow |
|  | 1677.0 | -7.085600,37.580952 | Fallow |

Notes: Low altitude=SUA, Medium altitude=Langali & High altitude=Nyandira
