# Supplementary material for: Elevation and land use shape soil entomopathogenic fungal communities in the Uluguru mountains, Tanzania: Insights from metagenomic and culture-based approaches
Source: PLoS One. 2026 May 11;21(5):e0348781. doi: 10.1371/journal.pone.0348781 (PMC13160300; doi:10.1371/journal.pone.0348781)
Supplement: S4 Table — (DOCX) [file pone.0348781.s004.docx]

**S4Table.** Normalized reads count assigned to specific EPF families isolated from different altitude and land use

| **Sample_ID** | | **Collection_site** | | **Altitude (m)** | | **Land_use** | | **Clavicipitaceae** | **Cordicipitaceae** | | **Ophiocordicipitaceae** | | **Bionecteriaceae** | | **Total nt_rpm** | **Total family/site** | | **%rpm/site** | |
| --- | --- | --- | --- | --- | --- | --- | --- | --- | --- | --- | --- | --- | --- | --- | --- | --- | --- | --- | --- |
| A | | SUA | | Low | | Cultivated | 3.7 | | 3.7 | | 7.4 | | 3.7 | 18.6 | | 4 | | 0.01 | |
| B | | SUA | | Low | | Cultivated | 0.0 | | 4.3 | | 0.0 | | 21.3 | 25.6 | | 2 | | 0.02 | |
| C | | SUA | | Low | | Fallow | 0.0 | | 81.0 | | 95.1 | | 0.0 | 176.2 | | 2 | | 0.11 | |
| D | | SUA | | Low | | Fallow | 77.0 | | 25.7 | | 25.7 | | 18.3 | 146.8 | | 4 | | 0.09 | |
| J | | Langali | | Medium | | Cultivated | 129.3 | | 79.9 | | 140.7 | | 3.8 | 353.7 | | 4 | | 0.23 | |
| I | | Langali | | Medium | | Cultivated | 144.5 | | 95.4 | | 159.0 | | 7.4 | 406.3 | | 4 | | 0.26 | |
| G | | Langali | | Medium | | Fallow | 31.1 | | 15.6 | | 84083 | | 3.9 | 84134 | | 4 | | 53.8 | |
| H | | Langali | | Medium | | Fallow | 47.6 | | 138.6 | | 43.3 | | 0.0 | 229.5 | | 3 | | 0.15 | |
| L | | Nyandira | | High | | Cultivated | 167.7 | | 4592 | | 1767 | | 52.2 | 6578 | | 4 | | 4.21 | |
| K | | Nyandira | | High | | Cultivated | 112.7 | | 4230 | | 995.1 | | 29.6 | 5368 | | 4 | | 3.44 | |
| E | | Nyandira | | High | | Fallow | 88.2 | | 0.0 | | 194.8 | | 7.3 | 290.3 | | 3 | | 0.19 | |
| F | | Nyandira | | High | | Fallow | 58329 | | 0.0 | | 187.4 | | 7.3 | 58523 | | 3 | | 37.5 | |
| **Total nt_rpm** | | | | | | | 59131 | | 9266 | | 87698 | | 155 | 156250 | |  | | 100 | |
| **%Ra/family** | | | | | | | 37.8% | | 5.9% | | 56.1% | | 0.1% | 100% | |  | |  | |
| **Total occurrence/family** | | | | | | | 10.0 | | 10.0 | | 11.0 | | 10.0 |  | |  | |  | |

Notes: Low=518m, Medium=1100m, High=1700m, Ra= Relative Abundance, rpm= reads per millions
