# Supplementary material for: Elevation and land use shape soil entomopathogenic fungal communities in the Uluguru mountains, Tanzania: Insights from metagenomic and culture-based approaches
Source: PLoS One. 2026 May 11;21(5):e0348781. doi: 10.1371/journal.pone.0348781 (PMC13160300; doi:10.1371/journal.pone.0348781)
Supplement: S5 Table — (DOCX) [file pone.0348781.s005.docx]

**S5Table.** Sequencing reads for EPF species across different samples

| **Sampling site** | **Altitude** | **Land_use** | ***M. anisopliae*** | ***P. lilacinum*** | ***C. rosea*** | ***P. chlamydosporia*** | ***H. thompsonii*** | ***M. suchlasporia*** | ***P. lavendulum*** | ***L. saksenae*** | ***M. pemphigi*** | ***C. javanica*** | ***A. lecanii*** | ***B. felina*** | ***Polycephalomyces sp.*** | ***Tolypocladium sp.*** | **Total rpm** |
| --- | --- | --- | --- | --- | --- | --- | --- | --- | --- | --- | --- | --- | --- | --- | --- | --- | --- |
| SUA | Low | Cu | 0.0 | 7.4 | 3.7 | 3.7 | 0.0 | 0.0 | 0.0 | 0.0 | 0.0 | 0.0 | 0.0 | 0.0 | 0.0 | 0.0 | 14.9 |
| SUA | Low | Cu | 0.0 | 0.0 | 21.3 | 0.0 | 0.0 | 0.0 | 0.0 | 0.0 | 0.0 | 4.3 | 0.0 | 0.0 | 0.0 | 0.0 | 25.6 |
| SUA | Low | Fa | 0.0 | 95.1 | 0.0 | 0.0 | 0.0 | 0.0 | 0.0 | 0.0 | 0.0 | 52.9 | 0.0 | 0.0 | 0.0 | 0.0 | 148.0 |
| SUA | Low | Fa | 14.7 | 7.3 | 18.3 | 62.4 | 0.0 | 0.0 | 3.7 | 0.0 | 0.0 | 0.0 | 0.0 | 25.7 | 0.0 | 14.7 | 146.8 |
| Langali | Medium | Cu | 91.3 | 102.7 | 3.8 | 30.4 | 15.2 | 0.0 | 0.0 | 3.8 | 7.6 | 3.8 | 0.0 | 0.0 | 0.0 | 0.0 | 258.6 |
| Langali | Medium | Cu | 57.5 | 31.1 | 0.0 | 0.0 | 0.0 | 0.0 | 0.0 | 0.0 | 48.6 | 0.0 | 0.0 | 0.0 | 0.0 | 0.0 | 137.3 |
| Langali | Medium | Fa | 0.0 | 84083.2 | 3.9 | 31.1 | 0.0 | 0.0 | 0.0 | 3.9 | 0.0 | 0.0 | 0.0 | 0.0 | 0.0 | 0.0 | 84122.1 |
| Langali | Medium | Fa | 4.3 | 34.6 | 0.0 | 13.0 | 0.0 | 0.0 | 0.0 | 0.0 | 30.3 | 0.0 | 0.0 | 0.0 | 0.0 | 0.0 | 82.3 |
| Nyandira | High | Cu | 85.7 | 37.3 | 52.2 | 29.8 | 0.0 | 11.2 | 0.0 | 0.0 | 41.0 | 3.7 | 3.7 | 0.0 | 11.2 | 0.0 | 275.8 |
| Nyandira | High | Cu | 8.7 | 0.0 | 7.3 | 22.2 | 0.0 | 14.7 | 0.0 | 0.0 | 0.0 | 0.0 | 0.0 | 0.0 | 0.0 | 0.0 | 52.9 |
| Nyandira | High | Fa | 88.2 | 172.7 | 0.0 | 0.0 | 0.0 | 0.0 | 0.0 | 0.0 | 0.0 | 0.0 | 0.0 | 0.0 | 0.0 | 0.0 | 260.9 |
| Nyandira | High | Fa | 66.0 | 188.7 | 0.0 | 0.0 | 0.0 | 58240.4 | 0.0 | 0.0 | 0.0 | 0.0 | 0.0 | 0.0 | 0.0 | 0.0 | 58495.1 |
| Total rpm/spp. | | | 416.3 | 84760.2 | 110.6 | 192.7 | 15.2 | 58266.3 | 3.7 | 7.7 | 127.5 | 64.7 | 3.7 | 25.7 | 11.2 | 14.7 | 144020.1 |

Notes: CU=Cultivated soils, Fa= Fallow soils, rpm= reads per millions,
