# Supplementary material for: Elevation and land use shape soil entomopathogenic fungal communities in the Uluguru mountains, Tanzania: Insights from metagenomic and culture-based approaches
Source: PLoS One. 2026 May 11;21(5):e0348781. doi: 10.1371/journal.pone.0348781 (PMC13160300; doi:10.1371/journal.pone.0348781)
Supplement: S6 Table — Mean estimates, lower (CI low) and upper (CI high) limits are shown for each site, grouped by elevation and land-use type. (DOCX) [file pone.0348781.s006.docx]

**S6Table.** Non-parametric bootstraps resampling estimates of Shannon alpha diversity (BCa 95% confidence intervals) for EPF communities across altitude × land-use combinations. Mean estimates, lower (CI low) and upper (CI high) limits are shown for each site, grouped by elevation and land-use type.

| **Altitude** | **Land use** | **Mean** | **CI low** | **CI high** |
| --- | --- | --- | --- | --- |
| High(1700m) | Cultivated | 1.496 | 1.291 | 1.496 |
| High(1700m) | Fallow | 0.335 | 0.031 | 0.335 |
| Low (518m) | Cultivated | 0.75 | 0.44 | 0.75 |
| Low (518m) | Fallow | 1.144 | 0.652 | 1.144 |
| Medium (1100m) | Cultivated | 1.308 | 1.166 | 1.308 |
| Medium (1100m) | Fallow | 0.587 | 0.004 | 0.587 |
