# Supplementary figures and images for: Elevation and land use shape soil entomopathogenic fungal communities in the Uluguru mountains, Tanzania: Insights from metagenomic and culture-based approaches
Source: PLoS One. 2026 May 11;21(5):e0348781. doi: 10.1371/journal.pone.0348781 (PMC13160300; doi:10.1371/journal.pone.0348781)

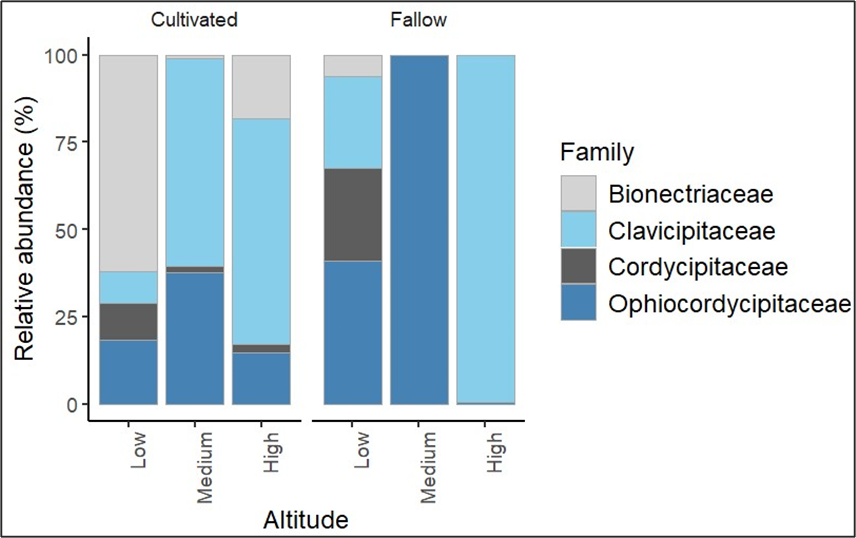

Supplement: S1 Fig — (TIF) [file pone.0348781.s007.tif]

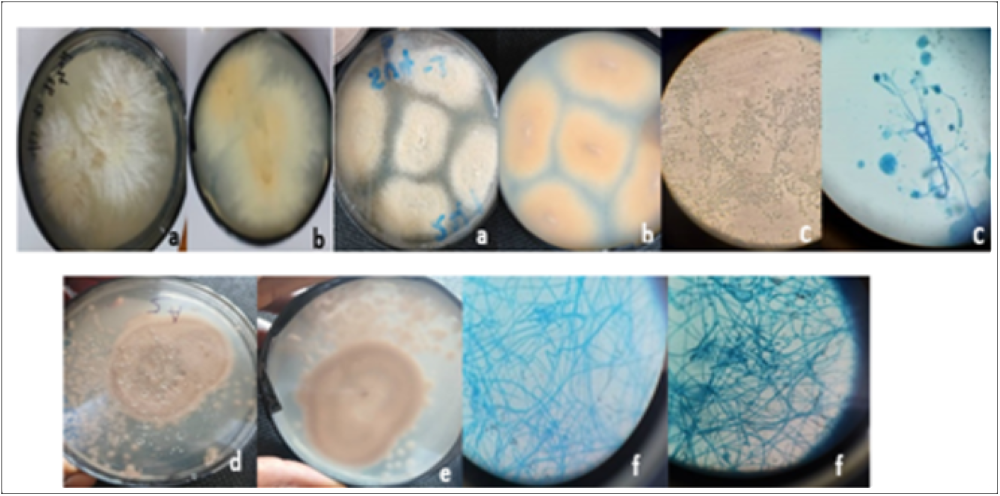

Supplement: S2 Fig — (TIF) [file pone.0348781.s008.tif]
