# Supplementary material for: Elevation and land use shape soil entomopathogenic fungal communities in the Uluguru mountains, Tanzania: Insights from metagenomic and culture-based approaches
Source: PLoS One. 2026 May 11;21(5):e0348781. doi: 10.1371/journal.pone.0348781 (PMC13160300; doi:10.1371/journal.pone.0348781)
Supplement: Attachment — Submitted filename: Response to Reviewers.docx [file pone.0348781.s010.docx]

**Rebuttal letter For Manuscript PONE-D-25-54696**

**Reviewer comment 1**

“Please ensure that your manuscript meets PLOS ONE's style requirements, including those for file naming. The PLOS ONE style templates for formatting sample main body and formatting sample title author affiliations.pdf”.

**Response 1:** We have carefully revised the manuscript to fully comply with PLOS ONE formatting and style requirements. The manuscript files have been correctly renamed s, and formatting has been standardized throughout (including Title and section headings, references, tables, and figure captions).

**Reviewer comment 2**

a). We note that the grant information you provided in the ‘Funding Information’ and ‘Financial Disclosure’ sections do not match. When you resubmit, please ensure that you provide the correct grant numbers for the awards you received for your study in the ‘Funding Information’ section.

b). Please provide an amended statement that declares *all* the funding or sources of support (whether external or internal to your organization) received during this study, as detailed online in our guide for authors at <http://journals.plos.org/plosone/s/submit-now>.  Please also include the statement “There was no additional external funding received for this study.” in your updated Funding Statement. Please include your amended Funding Statement within your cover letter. We will change the online submission form on your behalf.

c). We note that you have provided funding information that is currently declared in your Funding Statement. However, funding information should not appear in the Acknowledgments section or other areas of your manuscript. We will only publish funding information present in the Funding Statement section of the online submission form. Please remove any funding-related text from the manuscript and let us know how you would like to update your Funding Statement. Please include your amended statements within your cover letter; we will change the online submission form on your behalf.

**Response 2 a):** The previously stated grant number has been adjusted to resolve inconsistencies between the Funding Information and Financial Disclosure sections. The funding description has been harmonized across all relevant sections of the submission.

**Response 2 b):** The Funding Statement has been revised to fully declare that Sokoine University of Agriculture covered tuition and research costs only. The statement now includes: “There was no additional external funding received for this study.” The amended Funding Statement is included in the cover letter.

**Response 2 c):** All funding-related texts have been removed from the Acknowledgments and manuscript body. In addition, Data Availability statement, Author Contributions, Acknowledgments, and Competing Interests sections have been removed from the main manuscript. The revised Funding Statement is included in the cover letter for updating the submission form.

**Reviewer comment 3**

Please upload a new copy of Figure 7 as the detail is not clear. Please follow the link for more information: <https://journals.plos.org/plosone/s/figures>

**Response3:** A new high-resolution copy of Figure 7 has been prepared and uploaded according to the PLOS ONE figure guidelines to ensure clarity and readability.

**Reviewer comment 4**

Figure 1 in your submission contains map images which may be copyrighted. Please provide evidence that the figure can be published under CC BY 4.0 or replace it with a compliant map.

**Response 4:** We confirm that Figure 1 is an original map created by the authors using QGIS software (version 3.34.1) and does not include any copyrighted or proprietary basemaps (e.g., Google Maps or Google Earth). All spatial layers were derived solely from administrative boundary shapefiles obtained from the National Bureau of Statistics (NBS) of Tanzania, a public national institution, available via the NBS GIS portal (<https://www.nbs.go.tz/statistics/geographic-information-system-gis>). No satellite images or third-party proprietary data were used. Accordingly, Figure 1 fully complies with the Creative Commons Attribution (CC BY 4.0) license, and no additional permissions are required. The figure caption has been revised to reflect this (line 776-780).

**Reviewer comment 5**

The title accurately reflects the study focus. Consider including “metagenomic and culture-based approaches” to highlight methodological novelty. Abstract is concise and informative, but it overstates significance in places. For example, claims about “versatile taxa offering locally adapted candidates for biological control” should be tempered, as pathogenicity data are preliminary and limited to lab assays.

**Response 5:** The manuscript title has been revised to read “Elevation and land use shape soil entomopathogenic fungal communities in the Uluguru Mountains, Tanzania: insights from metagenomic and culture-based approaches” (lines 1-4). In the Abstract, statements regarding the potential of EPF taxa for biological control have been moderated (lines 58-60).

**Reviewer comment 6**

a). The introduction occasionally overgeneralizes (‘EPFs regulate more than 700 insect pests’) without specifying context (laboratory vs field). Consider clarifying.

b). The hypotheses are clear, but the expectation that fallow soils will always have higher diversity is not consistently supported in literature; the authors do acknowledge this later in discussion.

**Response 6 a):** We have revised the sentence in the Introduction to clarify the context as both laboratory, semi-field and field conditions (lines 53-54)

**Response 6b):** We note that we had already acknowledged this point in the Discussion (fourth paragraph; lines 421-423). We have further explained that the variability in diversity across fallow and cultivated from different publications (lines 431-434).

**Reviewer comment 7**

Sampling Design: Sampling is clearly described. The use of 24 soil samples is modest; this limits statistical power, especially for PERMANOVA (p ≈ 0.06). Consider discussing this limitation explicitly. In the results, the PERMANOVA finding (p = 0.06) should be described as a marginal trend and discussed in terms of ecological rather than strict statistical significance.

**Response 7:** To mitigate reduced statistical power associated with modest sample size, non-parametric bootstrapping was applied across alpha diversity, beta diversity, ordination, and prevalence analyses, enabling stable estimation of effect sizes and confidence intervals without reliance on large-sample assumptions, these have been incorporated in the results section (lines 339-343, 359-367, 484).

**Reviewer comment 8**

Quality control is thorough; however, the method for OTU clustering or ASV inference is not explicitly stated. Was DADA2, QIIME2, or another pipeline used? This is critical for reproducibility.
It is unclear how reads were assigned to EPF taxa. Were custom reference databases used for entomopathogenic fungi, or were general ITS databases applied? Misclassification of fungi is common in general ITS databases.

**Response 8:** This is now clarified in the data analysis section (lines 257-267);

Fungal reads assigned to known entomopathogenic fungi (EPF) were filtered and validated against a curated ITS reference dataset from GenBank. This ensures accurate species-level assignment and reduces mis-classification from general ITS databases, supporting reproducible analyses of EPF diversity, abundance, and phylogeny.

**Reviewer comment 9**

a). Larval bioassays are well-described. However, only 10 larvae per replicate (n=3) is a small sample size for mortality estimation, reducing statistical confidence. Consider larger replicates or a more robust statistical analysis.

b). Mortality range (10-50%) is broad; authors should clarify if variability is due to isolate differences or experimental conditions.

c). *P. lilacinum* and *C. rosea* show moderate virulence. The discussion rightly emphasizes ecological plasticity. However, the small-scale lab assay cannot directly support field-level pest management recommendations.

d) Acknowledgement of limitations on lack of direct link between soil chemistry/host density and EPF occurrence.

**Response 9 a):** We acknowledge that the bioassays used modest replication (10 larvae per replicate, n = 3), which limits statistical confidence. These were preliminary screenings to compare relative virulence among EPF isolates, summarized using mean ± SD, and interpreted cautiously. We have recommended that future studies should use larger sample sizes with increased replication for more robust laboratory and field validation (lines 209-211, 382-384, 466-477).

**Response 9 b):** The broad mortality range (10-50%) mainly reflects isolate-specific differences in virulence, with additional variation due to biological heterogeneity among larvae. This is consistent with previous EPF studies and suggests the need for isolate-specific and field-level validation (466-468).

**Response 9 c):** We have clarified in the Discussion that lab-based virulence results are preliminary and that field validation and lab dose-response pathogenicity are necessary before making pest management recommendations (lines 468-471).

**Response 9 d):** We added the limitation statement to indicate that single-season sampling and the absence of soil and host-density measurements limit mechanistic interpretation of EPF distribution and recommended future study to includes this (lines 471-477).

**Reviewer comment 10**

a). ITS-based phylogenies are acceptable for genus-level identification but have limited resolution at species level, especially for *Metarhizium* and *Purpureocillium*. Bootstrap values or support metrics in Fig. 7 are not discussed. Many entomopathogenic fungi require multi-locus markers (TEF1, RPB1/2) to resolve cryptic species; the manuscript should acknowledge this limitation.

b). Phylogenetic tree (Fig. 7) could be improved: Should include bootstrap values at all major nodes, label sequences with only accession numbers and species names (removing underlining, “.1”, and other symbols), incorporate an appropriate outgroup to root the tree, and be presented at a standard publication-quality level. Bootstrap values or support metrics should also be discussed in the text.

**Response 10 a):** We agree that ITS provides reliable genus-level resolution but limited discrimination for cryptic species, especially within *Metarhizium* and *Purpureocillium*. The manuscript now acknowledges this limitation, clarifying that ITS was used mainly for genus-level placement and preliminary species confirmation, and notes that multi-locus markers (e.g., TEF1-α, RPB1/2) are needed in future studies to resolve cryptic species (lines 445-450).

**Response 10 b):** Fig. 7 has been revised to include bootstrap values at all major nodes, label sequences using only GenBank accession numbers and species names (removing underlining, “.1”, and extra symbols), and incorporate an appropriate outgroup to properly root the tree. The figure has been formatted to a standard publication-quality level, and the revised manuscript now discusses bootstrap support in the Results section.

**Reviewer comment 11**

**a)** Observation of “hump-shaped” diversity at mid-elevation is interesting, but limited replication and low sample size may affect robustness. Relative abundance discussion is clear but could be supported with formal statistical testing (e.g., differential abundance analysis using DESeq2 or ANCOM).

**b)** Some statements about ecological implications (e.g., “medium elevation zones are biodiversity hotspots”) are plausible but need cautious phrasing given limited data.

**Response 11 a):** We acknowledge that limited replication and modest sample sizes may affect the observed mid-elevation “hump-shaped” pattern. Non-parametric bootstrapping was applied to diversity and prevalence analyses to provide stable effect estimates. While formal differential abundance testing (e.g., DESeq2, ANCOM) was not performed, the bootstrapping supports the observed relative abundance patterns (lines 483-492).

**Response11 b):** We have revised the phrasing to reflect caution. The text now states that medium-elevation habitats may represent areas of relatively elevated diversity within the sampled gradient, and we note that broader spatial and temporal sampling is required before designating them as definitive biodiversity hotspots (lines 415-418).

**Reviewers comment 12**

Table 3 could report standard deviation of larval mortality to better illustrate variability.

**Response 12:** Table 3 has been updated to report mean mortality ± SD, and observed mortality range, to clearly show variability.

**Reviewer comment 13**

Suggestions to clarify on: a) Grammatical and references improvement including Correct spelling of “Purpureocillim”to “Purpureocillium” scientific names italicizing in multiple places. b) Improve figure labeling for clarity (colors, symbols, axis labels). c) Include standard deviations or error bars for mortality assays. d) Clarify whether fallow soils were recently abandoned or long-term fallows, as this affects fungal diversity. e) Verify that all figures and tables (1-7) are cited in sequence and that each caption is descriptive and self-contained. Sampling is clearly described. Consider discussing this limitation explicitly.

**Response 13:** We thank the reviewer for these suggestions. (a) The manuscript has been carefully revised to improve grammar, spelling, formatting and clarity including *Purpureocillim* to *Purpureocillium*, all scientific names have been italicized, and both the in-text citations and reference list have been revised to ensure accuracy and compliance with PLOS ONE guidelines. (b) Figure labeling has been improved for clarity, including colors, symbols, and axis labels. (c) Mortality data now include mean ± SD in table 3. (d) We have clarified in the Methods that fallow soils were left uncultivated at least 2 years, noting potential implications for fungal diversity (lines 155-157). e) all figures and tables (1-7) have been verified to ensure that they are cited in a sequence, and captions have been revised to be descriptive and self-contained.
