# Supplementary material for: Elevation and land use shape soil entomopathogenic fungal communities in the Uluguru mountains, Tanzania: Insights from metagenomic and culture-based approaches
Source: PLoS One. 2026 May 11;21(5):e0348781. doi: 10.1371/journal.pone.0348781 (PMC13160300; doi:10.1371/journal.pone.0348781)
Supplement: Attachment — Submitted filename: Response_to_Reviewers_auresp_2.docx [file pone.0348781.s011.docx]

**Rebuttal letter For Manuscript PONE-D-25-54696R1**

We thank the reviewer for these constructive suggestions. The following are the responses to each point raised.

**Reviewer comment 1**

Bioinformatics approach: While the use of CZ ID for taxonomic classification is clearly described, it would be helpful if the authors briefly acknowledge in the discussion that the absence of ASV/OTU-based inference may influence fine-scale diversity estimates. This clarification would strengthen transparency but does not affect the overall conclusions.

**Response 1:** We have now included in the Discussion (lines 484-489) a note acknowledging that lack of ASV/OTU-based inference may limit resolution of fine-scale diversity patterns and future studies could integrate ASV/OTU-based approaches, while emphasizing that this does not affect the broader ecological interpretations.

**Reviewer comment 2**

Interpretation of diversity patterns: The observation that cultivated soils sometimes show higher diversity than fallow soils is interesting. A short note emphasizing that this pattern may be context-dependent (e.g., influenced by local agricultural practices or soil properties) would improve interpretation.

**Response 2:** We have added a note in the Discussion (lines 437-439) emphasizing that the observed higher diversity in cultivated soils is likely context-dependent and may be influenced by local agricultural practices, soil properties, and management intensity.

**Reviewer comment 3**

Pathogenicity assay: The authors have appropriately described these experiments as preliminary. For clarity, they may briefly mention in the discussion that future studies could include dose-response assays and larger sample sizes to better quantify virulence

**Response 3:** This point was already addressed in the Discussion (lines 472-479), where we noted that the assays were preliminary, acknowledged the limited number of replications and highlighted the need for increased replication, larger sample sizes, and dose-response assays in future lab studies. However, to improve readability and clarity, we have now split the long sentence into two (lines 476-481); no new content was added.

**Reviewer comment 4**

Minor technical corrections: Check for minor typographical issues (e.g., “OUT” vs “OTU”). Ensure consistency in reporting statistical values and terminology.
A quick check of PCR conditions (e.g., extension temperature) for clarity

**Response 4:** We have carefully checked the manuscript for typographical errors (e.g., corrected “OUT” to “OTU”; line 264, *Lecanicilium* to *lecanicillium*; line 316 & Table 2), ensured consistent reporting of statistical values and terminology throughout the manuscript, and PCR conditions have been adjusted for clarity (lines 232-234).

**Reviewer Comment 5**
Please verify that all references are correct and ensure that no retracted articles are cited. If any retracted articles are included, provide justification or replace them with appropriate references.

**Response 5:** We carefully reviewed the entire reference list and cross-checked all citations using reliable academic sources. We confirm that none of the cited articles have been retracted; therefore, no removals or replacements were necessary. The references were initially formatted using Zotero based on the PLOS style, and minor inconsistencies were corrected manually (ref 47, line 468, ref 20, line 593 was CAB abstract not a primary publication now has been replaced with original publication.
