# Supplementary material for: Elevation and land use shape soil entomopathogenic fungal communities in the Uluguru mountains, Tanzania: Insights from metagenomic and culture-based approaches
Source: PLoS One. 2026 May 11;21(5):e0348781. doi: 10.1371/journal.pone.0348781 (PMC13160300; doi:10.1371/journal.pone.0348781)
Supplement: Attachment — Submitted filename: Response_to_Reviewers_auresp_3.docx [file pone.0348781.s012.docx]

**Rebuttal letter For Manuscript PONE-D-25-54696R2**

We thank the Editor/Reviewer for the constructive suggestions. The following is the responses to point raised.

**Comment**

The authors must include the BioProject and SRA numbers of the sequences into the manuscript at the materials and methods, where relevant.

**Response:** The BioProject accession number (PRJNA1425464) and corresponding BioSample accessions (SAMN55416426-SAMN55416435) have now been explicitly included in the Materials and Methods section under the high-throughput sequencing subsection (lines 183-187). Although these details were already provided in the online submission system’s Data Availability statement, we have now also incorporated them directly into the manuscript text to improve clarity and ensure compliance with journal requirements.
